# FOXA1 is a determinant of drug resistance in breast cancer cells

**DOI:** 10.1007/s10549-020-06068-5

**Published:** 2021-01-08

**Authors:** Uttom Kumar, Anastasia Ardasheva, Zimam Mahmud, R. Charles Coombes, Ernesto Yagüe

**Affiliations:** 1 Division of Cancer, Imperial College Faculty of Medicine, Hammersmith Hospital Campus, Du Cane Road, London, W12 0NN UK; 2 Present Address: Medical Sciences Division, University of Oxford, Oxford, UK; 3 Present Address: Department of Biochemistry and Molecular Biology, University of Dhaka, Dhaka, 1000 Bangladesh

**Keywords:** FOXA1, E-cadherin, Multidrug resistance, Breast cancer, Anchorage independence

## Abstract

**Purpose:**

Breast cancer is one of the most commonly diagnosed cancers in women. Five subtypes of breast cancer differ in their genetic expression profiles and carry different prognostic values, with no treatments available for some types, such as triple-negative, due to the absence of genetic signatures that could otherwise be targeted by molecular therapies. Although endocrine treatments are largely successful for estrogen receptor (ER)-positive cancers, a significant proportion of patients with metastatic tumors fail to respond and acquire resistance to therapy. FOXA1 overexpression mediates endocrine therapy resistance in ER-positive breast cancer, although the regulation of chemotherapy response by FOXA1 has not been addressed previously. FOXA1, together with EP300 and RUNX1, regulates the expression of E-cadherin, and is expressed in luminal, but absent in triple-negative and basal-like breast cancers. We have previously determined that EP300 regulates drug resistance and tumor initiation capabilities in breast cancer cells.

**Methods:**

Here we describe the generation of breast cancer cell models in which FOXA1 expression has been modulated either by expression of hairpins targeting *FOXA1* mRNA or overexpression plasmids.

**Results:**

Upon FOXA1 knockdown in luminal MCF-7 and T47D cells, we found an increase in doxorubicin and paclitaxel sensitivity as well as a decrease in anchorage independence. Conversely, upregulation of FOXA1 in basal-like MDA-MB-231 cells led to an increase in drug resistance and anchorage independence.

**Conclusion:**

Together, these data suggest that FOXA1 plays a role in making tumors more aggressive.

**Supplementary Information:**

The online version contains supplementary material available at 10.1007/s10549-020-06068-5.

## Introduction

Breast cancer is the most common cancer in women, affecting approximately 2 million women each year worldwide. In 2018 alone, breast cancer accounted for 15% of all cancer-related deaths in women, claiming lives of over 600,000 women worldwide [1]. Based on tumor genetic profiling, five main molecular breast cancer subtypes have been defined: luminal A, luminal B, triple-negative/basal-like, HER2-enriched, and normal-like [2]. These do not only differ in their gene expression signatures, but also carry different prognostic values. Thus, luminal A tumors are estrogen receptor (ER)-positive and have favorable clinical prognosis, while triple-negative/basal-like subtype tumors are challenging to treat due to a lack of targeted therapies [1]. The molecular classification of breast cancer was introduced 20 years ago and since then several new subtypes have been included, such as claudin-low (characterized by the low expression of E-cadherin, high expression of epithelial-to-mesenchymal transition (EMT) genes, and expression of stem cell-like genetic signatures) [3] or molecular apocrine breast cancer (an aggressive form of ER-negative breast cancer which expresses androgen receptor and has a poor clinical prognosis) [4]. Therefore, there is a large ongoing effort in the field to identify novel molecular targets that would allow treatment of a broad range of breast tumors.

The Forkhead box (FOX) protein family encompasses over fifty transcriptional regulators and is characterized by the presence of the highly conserved “forkhead” DNA-binding domain. These proteins play a role in a multitude of biological processes, such as development, proliferation, and longevity [5]. Forkhead proteins FOXA, FOXC, FOXM, FOXO and FOXP are important players in oncogenic and tumor suppression pathways [6, 7]. Upregulation of FOXM1 and members of the FOXC family is common in multiple carcinomas, such as those from the lung, prostate, pancreas, and breast, and is associated with uncontrolled cell proliferation, metastasis, and poor clinical prognosis, illustrating their oncogenic role [8, 9]. In contrast, members of the FOXO family act as tumor suppressors and negatively regulate cell proliferation and survival. Thus, FOXO3a negatively regulates interferon-dependent genes, which are important for metastasis, and acts as an antagonist to oncogenic FOX proteins [10, 11]. In prostate cancer, the role of FOXA1 is controversial, as both reports on its role as an oncogene and metastasis suppressor have been published [12, 13]. As FOX proteins can drive or suppress oncogenesis, understanding their role in malignant transformation is crucial for the development of novel therapeutic strategies.

FOXA1 is expressed in all luminal breast cancer cell lines, all ER-positive tumors, and about half of ER-negative tumors [14]. In ER-positive tumors, FOXA1 expression carries a favorable clinical outcome as FOXA1 is a pioneer factor, opening chromatin and allowing ER to interact with its response elements and mediate response to endocrine therapies, such as tamoxifen [15]. While endocrine treatments are largely successful, about half of patients with metastatic tumors fail to respond and almost all relapse due to acquiring resistance to therapy [16]. One of the mechanisms underlying drug resistance in ER-positive tumors is the reprogramming of ER activity triggered by cross-talk with growth factor receptor pathways. FOXA1 is considered one of key modulators of this reprogramming in ER-positive therapy-resistant tumors as it modulates the expression levels of ER-regulated genes, some of which, in turn, can make a tumor more aggressive [17]. Thus, one of the genes upregulated by FOXA1, IL-8, promotes tumor survival, metastasis, and is associated with resistance to ER inhibitors [18]. These findings emphasize the importance of understanding the role of FOXA1 further and exploring the potential of using it as a therapeutic target in ER-positive breast tumors.

FOXA1, together with EP300 and RUNX1, promotes the expression of E-cadherin [19], which, in turn, reduces the ability of cancer cells to trigger an EMT and initiate metastasis [20]. EMT, together with the acquisition of drug resistance and stem-like properties, are some of the processes known to be associated with tumor progression [21]. It is well established a role for FOXA1 in endocrine therapy resistance [18]; however, its association with chemotherapy response has not been established. In this study we have experimentally modulated FOXA1 levels in breast cancer cell models and determined its role in drug resistance and tumor formation in vitro*.*

## Materials and methods

### Cells

European Cell Culture Collection cell lines MCF-7, T47D, MDA-MB-231 and HS578T were obtained from Sigma-Aldrich. CAL51 cells were obtained from the German Resource Centre for Biological Material (DSMZ). These cells were maintained in Dulbecco’s Modified Eagle Medium (DMEM) supplemented with 1 g/L glucose, 10% fetal calf serum and 4 mM l-glutamine (Thermo Fisher Scientific). Medium for HS578T cells contained, in addition, 10 µg/mL recombinant human insulin (Sigma-Aldrich). MCF-10A cells were a gift from L. Buluwela (Imperial College London) and were routinely cultivated on serum-free HuMEC medium (Thermo Fisher Scientific). Where indicated, cells were treated with 5-azacytidine (azaC; Sigma-Aldrich) daily for 3 days.

### Experimental modulation of FOXA1 expression

Overexpression of *FOXA1* was obtained by stable expression of pcDNA3.1(−)-derived constructs carrying the full-length cDNA. *FOXA1* cDNA was synthesized by GenScript Biotech and cloned into the *Xho*I and *Eco*RI sites of pcDNA3.1(−) (Thermo Fisher Scientific) incorporating a GCCACC Kozak sequence immediately before the ATG initiation codon. Plasmid DNA was linearized with *Pvu*I and transfected into MDA-MB-231 cells using FuGENE 6 (Promega) following manufacturer's instructions (16 µg DNA at 1:1.5 DNA: FUGENE ratio in 500 µL Opti-MEM, Thermo Fisher Scientific, for 1.5 × 10^6^ cells in 25 cm^2^ flasks) and selected and maintained with 1 mg/mL G418 (Sigma-Aldrich). Downregulation of *FOXA1* in MCF-7 and T47D cells was obtained by stable expression of hairpins in the lentiviral vector pGIPZ (Horizon Discovery). Six different hairpins targeting *FOXA1* mRNA (RNAi Consortium, Broad Institute) were used (clones id and mature antisense sequences were sh1: V2LHS_16813, 5′-TTATGGTTAAGAGTATTGC-3′; sh2: V3LHS_414340, 5′-ATAACAGCAGTATTGTCTT-3′; sh3: V3LHS_399489, 5′-TCTCGAACATGTTGCCGGA-3′; sh4: V3LHS_414344, 5′-GTCTTGCTATATAACAGCA-3′; sh5: V3LHS_414341, 5′-ATAACTTATCTCTCCTCCA-3′; sh6: V2LHS_16780, 5′-GTTATGTAAATATACGGAG-3′). Viral transductions were performed essentially as described [22] and cells were selected and maintained with 1 µg/mL puromycin.

### Gene expression analysis

Total RNA, isolated using a RNeasy Mini kit (Qiagen) was reverse transcribed using a High Capacity cDNA Reverse Transcription Kit (Applied Biosystems) and real-time quantitative PCR was performed using PowerUp SYBR Green Master Mix (Applied Biosystems) on a StepOnePlus Real-Time PCR system (Applied Biosystems). The cycling program included a 90 °C for 10 min initial step for enzyme activation followed by 40 cycles of denaturation and primer annealing/extension consisting of 95 °C for 3 s and 60 °C for 30 s, respectively. The ribosomal RPS14 and RPLP0 genes were used as normalizers. Primer sequences are described in Supplementary Table 1. A comparative threshold cycle was used to determine relative gene expression with standard curves for each gene as previously described [23].

### Immunoblotting

Antibodies for immunodetection following standard immunoblotting procedures were 24E10 for E-cadherin (Cell Signaling Technology), Q6 (Santa Cruz Biotechnology) for FOXA1 and AC-15 (ab6276; Abcam) for β-actin. Primary antibodies were detected using horseradish peroxidase linked anti-mouse or anti-rabbit secondary antibodies as appropriate (Dako), and visualized using the ECL detection system (Amersham Biosciences). Imaging was performed using public domain ImageJ software (National Institutes of Health, https://imagej.nih.gov/ij/).

### Drug sensitivity assay

A sulforhodamine B (SRB, Sigma-Aldrich) assay [24] was used to screen for drug cytotoxicity essentially as previously described [25]. Briefly, 2 × 10^3^ cells were plated per well of a 96-well plate and the following day treated with serial dilutions of the corresponding drug for a length of time equivalent to two doubling times. Drugs used were doxorubicin (Pharmachemie B.V.) and paclitaxel (Tocris). Cells were then fixed with cold 40% (w/v) trichloroacetic acid (Sigma-Aldrich) for 1 h at 4 °C, washed, stained with 0.4% (w/v) SRB in 1% (v/v) acetic acid for 1 h at room temperature, washed with 1% (v/v) acetic acid, and dried overnight. The next day, the dye was solubilized with 10 mM Tris (pH 7) for 1 h at room temperature, and optical density was measured at 490 nm using a Sunrise microplate reader (Tecan). LD_50_ was considered as the drug concentration that killed 50% of cells from best-fit curves generated using Graphpad Prism 6 (Graphpad Software Inc.).

### Anchorage-independent growth assays

For soft agar assays, 2 × 10^4^ cells were mixed with DMEM containing 0.3% agar noble (Difco Laboratories) on 6-well plates with a bottom layer of solidified 0.6% agar noble in the same medium. Triplicate cultures for each cell type were maintained for 4 weeks at 37 °C in an atmosphere of 5% CO_2_ and 95% air, with 300 μL fresh medium added twice a week. After 4 weeks, colonies of at least 100 μm in diameter were counted. For mammosphere formation assays, 4 × 10^3^ cells/well were seeded, in triplicate, in ultra-low-attachment 6-well plates (Corning) in DMEM/F12 medium supplemented with 20 ng/mL epidermal growth factor (EGF, Sino Biologicals), 20 ng/mL basic fibroblast growth factor (bFGF, Thermo Fisher Scientific) and B27 (Thermo Fisher Scientific). Fresh medium (500 μL) was added every 3–5 days for 2 weeks after which the spherical clusters of cells were counted. Clone or sphere forming efficiency was calculated relative to the total number of plated cells 100×. Images were captured using an EVOS FL Cell Imaging System (Thermo Fisher Scientific).

### Statistical analysis

Statistical evaluations were performed by Student’s *t* test for paired data. Data were considered significant at a *p*-value inferior to 0.05.

## Results

### Modulation of FOXA1 in cell model systems

**Fig. 1:**
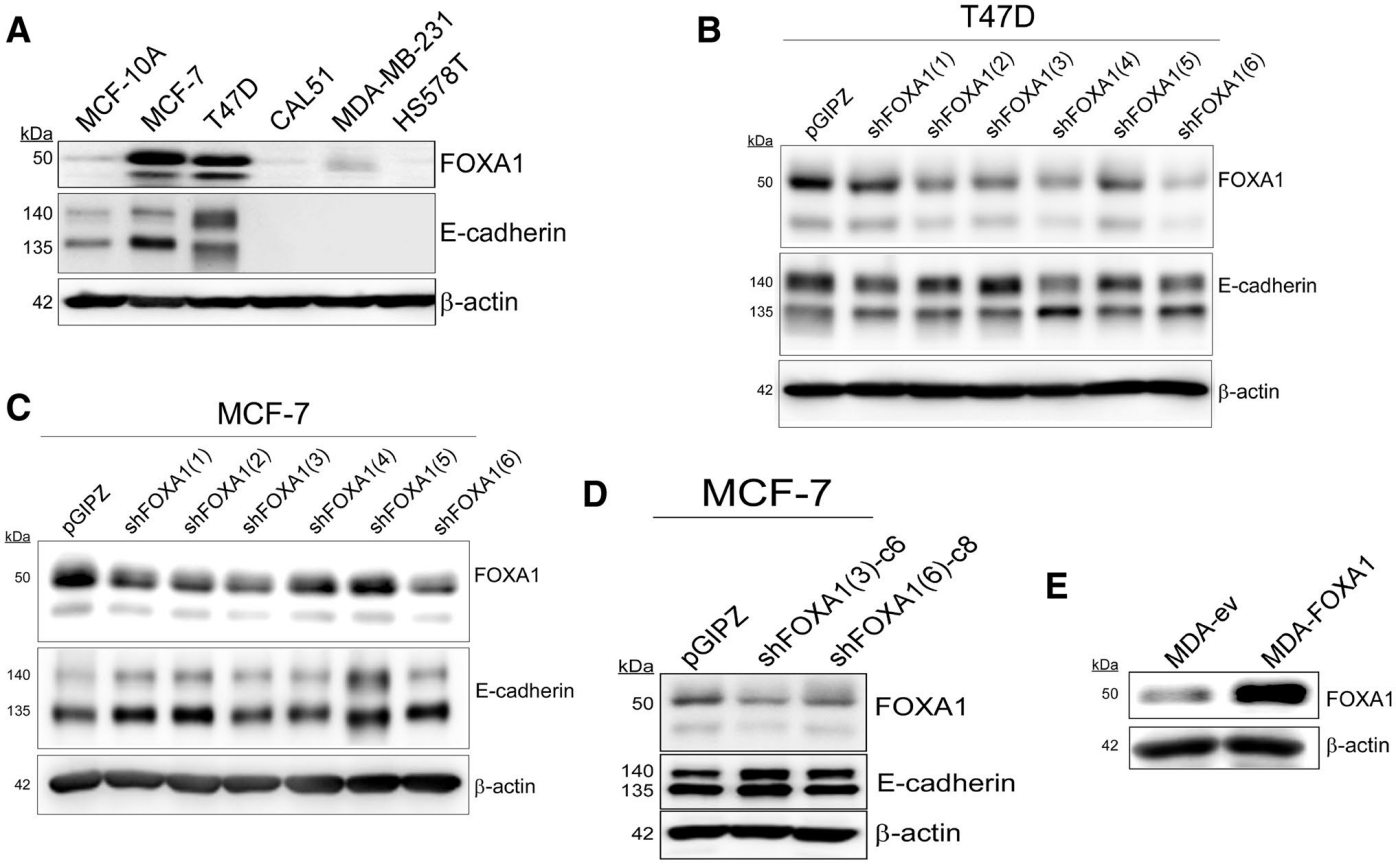
Experimental modulation of FOXA1 in breast cancer cell lines. **a** FOXA1 is highly expressed in luminal cells. Western blot of FOXA1 and E-cadherin in a small panel of breast cancer cell lines. E-cadherin is detected as two bands, 140 kDa precursor and 135 kDa after convertase processing. Generation of stably transfected T47D (**b**) and MCF-7 (**c**) cells by expression of *FOXA1* mRNA targeting small hairpins (shFOXA1(1) to shFOXA1(6)) and detection of FOXA1 and E-cadherin levels by western blot. Empty vector pGIPZ was used as a negative control. **d** Selection of clones from MCF-7 transfected pools. The two clones with the most FOXA1 downregulation were selected and its expression detected by western blot analysis. **e** Generation of stably transfected MDA-MB-231 cells by transfection of a FOXA1-expressing plasmid and detection by western blot. Empty pcDNA3.1 vector (ev)-transfected cells were used as a control. In all immunoblots β-actin was used as a loading control. Molecular masses of relevant bands are indicated in kDa

In order to study how breast cancer cells respond to experimental FOXA1 modulation, we first determined FOXA1 expression levels in a small panel of breast cancer cell lines. Immunoblot analysis indicated that, as expected, FOXA1 was expressed by luminal cells such as MCF-7 and T47D cell lines. The three basal-like, triple-negative cell lines used, CAL51, MDA-MB-231, and HS578T, did not express FOXA1, although a faint signal was obtained in MDA-MB-231 cells. The non-tumorigenic cell line MCF-10A also showed a faint signal in immunoblots (Fig. 1a). FOXA1 is a transcriptional activator of *CDH1*, the gene encoding E-cadherin [19], and a positive correlation was observed between the expression of both proteins. MCF-7 and T47D cells showed elevated E-cadherin, MCF-10A cells showed intermediate, and triple-negative cell lines absent expression (Fig. 1a). The *CDH1* promoter in MDA-MB-231 cells is methylated, and therefore the absence of E-cadherin expression despite low, but detectable, levels of FOXA1 probably reflects the closed chromatin configuration of the *CDH1* locus.

We generated stably transfected MCF-7 and T47D cells with a lentivirus driving the expression of hairpins targeting *FOXA1* mRNA. FOXA1 expression was high in T47D cells transfected with the empty lentiviral vector and decreased to varying extents upon expression of six different hairpins (Fig. 1b). Image quantification indicated that the two best hairpins, sh6 and sh4, produced FOXA1 downregulation by approximately 90% and 65%, respectively (Supplementary Fig. 1a). Cells generated with these two hairpins were selected for further studies (hereby named T47D-shFOXA1(6) and T47D-shFOXA1(4) cells, respectively). Equally, MCF-7 control cells transfected with the empty lentivector showed a high level of FOXA1 expression. Transfection with the same six hairpins targeting *FOXA1* mRNA did not show a high level of downregulation as in the case of T47D cells (Fig. 1c and Supplementary Fig. 1b), and these cells were not considered suitable for further biological analysis as pools. Thus, individual clones were isolated from the pools of cells generated with the two best hairpins, sh3 and sh6. The best FOXA1 downregulating clone from each hairpin was selected (clone 6 for hairpin 3 and clone 8 for hairpin 6, hereby named MCF7-shFOXA1(sh3)-c6 and MCF7-shFOXA1(sh6)-c8, respectively) (Fig. 1d) for further studies. These cells showed downregulation of FOXA1 levels by 60%, MCF7-shFOXA1(sh3)-c6, and 50%, MCF7-shFOXA1(sh6)-c8 (Supplementary Fig. 1c). Triple-negative, basal-like breast cancer cells show many mesenchymal characteristics and thus we generated stably transfected cells derived from MDA-MB-231 cells in which FOXA1 was upregulated after transfection with an expression vector. MDA-MB-231 cells expressed low levels of FOXA1 that were upregulated approximately threefold in the stably over-expressed cells (Fig. 1e and Supplementary Fig. 1d).

### Experimental modulation of FOXA1 in breast cancer cells does not affect E-cadherin levels

**Fig. 2:**
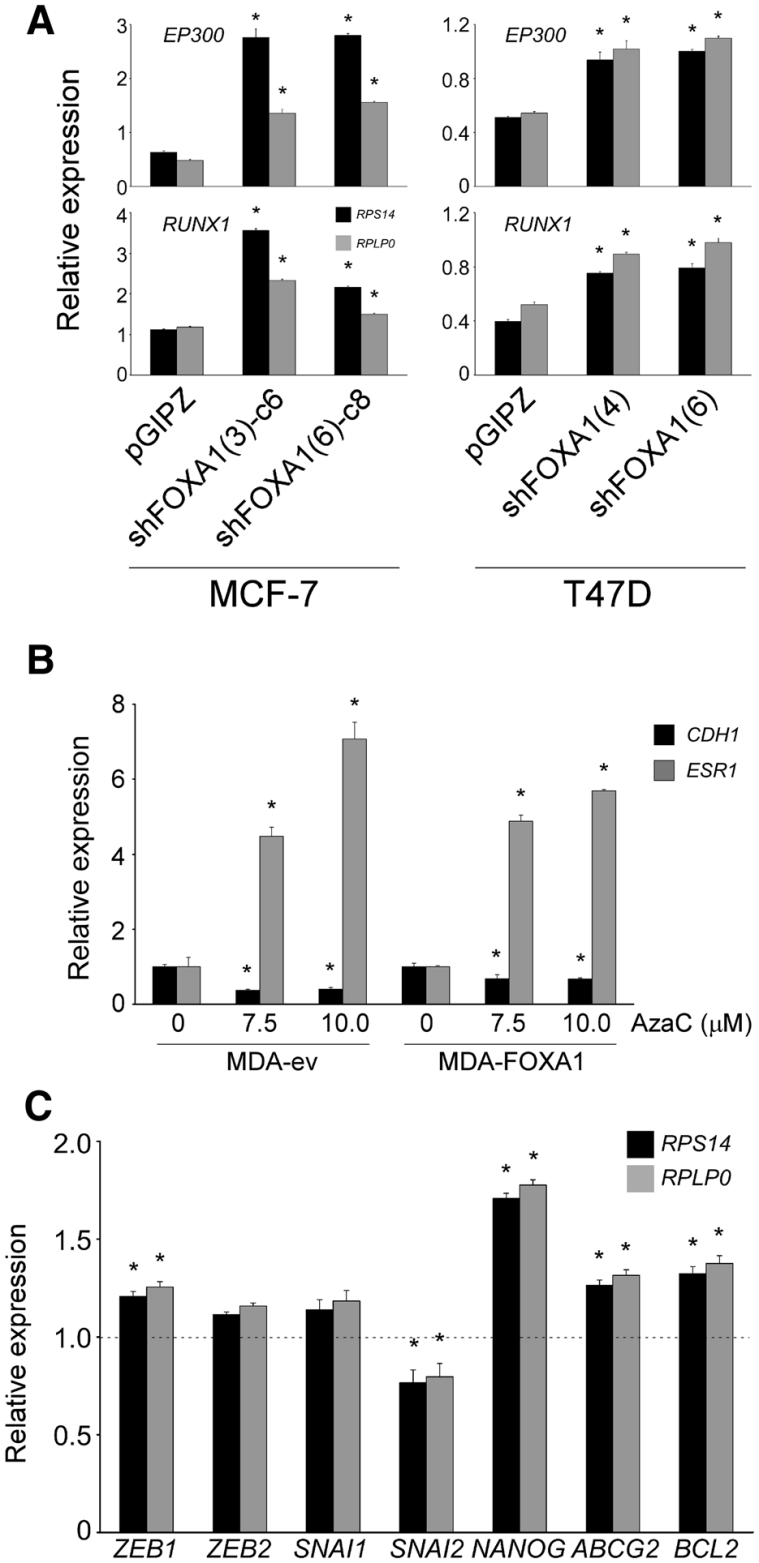
Gene expression analysis of FOXA1-modulated breast cancer cells. **a** Transcriptional activators of E-cadherin expression are upregulated upon experimental knockdown of FOXA1. Expression analysis by real-time quantitative PCR of *EP300* (top panels) and *RUNX1* (bottom panels) in FOXA1-knocked down MCF-7 (left panels) and T47D (right panels) cells. The ribosomal *RPS14* and *RPLP0* genes were used as normalizers. **b** Overexpression of FOXA1 in MDA-MB-231 cells is unable to trigger E-cadherin expression after DNA demethylation. Cells were treated daily with the demethylating agent AzaC for 3 days and *CDH1* expression was determined by real-time quantitative PCR. Activation of *ESR1* expression was used as a control for azaC treatment. *RPS14* was used as a normalizer. Data indicate the average ± SD of three replicates. *p < 0.05. **c** Differential regulation of key genes upon FOXA1 upregulation in MDA-MB-231 cells. Gene expression was determined in MDA-FOXA1 cells by real-time quantitative PCR. The ribosomal *RPS14* and *RPLP0* genes were used as normalizers. Data indicate the average ± SD of three replicates. *p < 0.05 after comparing to the corresponding expression values from control MDA-ev cells (dashed line)

Interestingly, E-cadherin levels did not decrease upon downregulation of FOXA1 in luminal MCF-7 and T47D cells (Fig. 1b, d). Blot quantification showed that some hairpins did indeed reduced E-cadherin expression in T47D cells, but this was only by approximately 10% (Supplementary Fig. 1a). In MCF-7 cells there was even a slight increase in E-cadherin expression (Supplementary Fig. 1b, c). This lack of E-cadherin downregulation upon FOXA1 knockdown probably reflects the fact that cells did overcompensate with an upregulation of the other two transcriptional activators of E-cadherin, EP300 and RUNX1 (Fig. 2a). Overexpression of FOXA1 in MDA-MB-231 cells did not upregulate the practically absent E-cadherin levels at either protein or mRNA level (data not shown). As MDA-MB-231 cells have a highly methylated *CDH1* locus [26], FOXA1-overexpressing MDA-MB-231 cells were treated with the demethylating agent azaC. Indeed, demethylation of DNA activated the transcription of *ESR1*, which was used as a positive control for azaC treatment [27], although it did not trigger activation of the *CDH1* locus (Fig. 2b). Although MDA-FOXA1 cells showed a slight increase in E-cadherin transcriptional repressor *ZEB1* mRNA levels, others, such as *ZEB2* and *SNAI* did not change, and *SNAI2* was slightly downregulated (Fig. 2c). Thus, although *ZEB1* might contribute to the lack of E-cadherin expression in MDA-FOXA1 cells, these data suggest a deeper hierarchy in the FOXA1 control on the *CDH1* locus. Importantly, these cell models represent good systems to analyze the phenotypic effects of FOXA1 independently of E-cadherin.

### FOXA1 levels determine the sensitivity of breast cancer cells to chemotherapeutic drugs

**Fig. 3:**
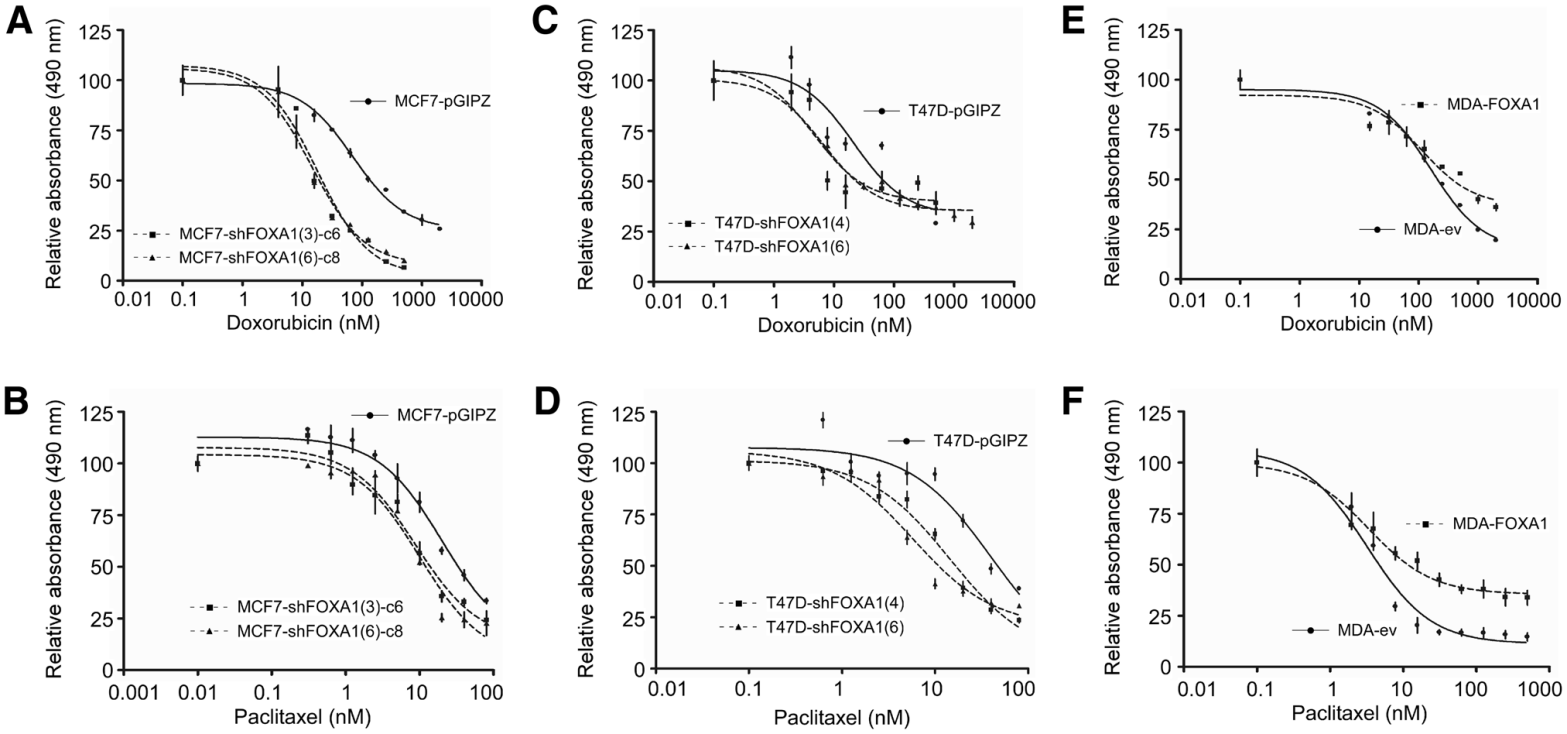
Experimental modulation of FOXA1 levels affects drug sensitivity. Drug sensitivity curves obtained with doxorubicin (*upper panels*) and paclitaxel (*lower panels*) in FOXA1-downregulated MCF-7 (**a**, **b**), T47D (**c**, **d**) and FOXA1-overexpressing MDA-MB-231 (**e**, **f**) cells. Data points represent the average ± SD from six replicates and the best-fit curves are shown

Next, we determined how an experimental variation in FOXA1 expression levels would affect the sensitivity of breast cancer cells to drugs commonly used in cancer chemotherapy. For this we selected doxorubicin, an anthracycline commonly used in many combination regimes, which acts on topoisomerase II, and paclitaxel, a microtubule poison, used as part of the armamentarium for metastatic breast cancer. MCF-7 cells in which FOXA1 was downregulated showed an important increase in sensitivity to doxorubicin (Fig. 3a). Cells generated with two different hairpins showed similar increases in sensitivity (sixfold to sevenfold, Table 1). Response to paclitaxel was also toward an increase in sensitivity (Fig. 3b), although with this drug the increase in sensitivity did not reach twofold (Table 1). In a similar fashion, T47D cells responded to both drugs with an increase in sensitivity, and, as in MCF-7 cells, both hairpins showed a similar trend (Fig. 3c, d). Doxorubicin sensitivity increased between 2.0 and 2.8-fold, whereas paclitaxel sensitivity increased between 1.8 and 3.2-fold (Table 1). Conversely, experimental overexpression of FOXA1 in MDA-MB-231 cells led to increases in doxorubicin and paclitaxel resistance by 1.8- and 2.1-fold, respectively (Fig. 3e, f; Table 1), leading to a multidrug resistance phenotype [28].

**Table 1:** Sensitivity of FOXA1-modulated cells to doxorubicin and paclitaxel

Cell	FOXA1 modulation	LD_50_ (nM)		Resistance ratio^a^	
		Doxorubicin	Paclitaxel	Doxorubicin	Paclitaxel
MDA-ev		199.8	5.5		
MDA-FOXA1	Upregulation	364.8	11.7	1.8	2.1
MCF7-pGIPZ		128.0	28.6		
MCF7-shFOXA1(3)-c6	Downregulation	20.2	15.2	− 6.3	− 1.9
MCF7-shFOXA1(6)-c8	Downregulation	18.3	16.5	− 7.0	− 1.7
T47D-pGIPZ		53.0	32.6		
T47D-shFOXA1(4)	Downregulation	26.5	18.1	− 2.0	− 1.8
T47D-shFOXA1(6)	Downregulation	19.0	10.0	− 2.8	− 3.2

^a^Positive values indicate resistance; negative values indicate sensitivity

As P-glycoprotein (ABCB1) is one of the main mechanisms by which acquire resistance to structurally and mechanistically different drugs, we attempted to measure *ABCB1* expression in MDA-FOXA1 cells by real-time quantitative PCR, but the signals were below detection levels (data not shown). Equally, MCF-7 and T47D cells do not express ABCB1 [29]. Another two genes involved in drug resistance, *ABCG2*, encoding another ABC transporter, and the anti-apoptotic *BCL2*, increased slightly their expression levels (1.3–1.4 fold, Fig. 2c), although is unlikely that these small variation will be totally responsible for the LD_50_ changes associated with FOXA1 overexpression [30, 31].

Thus, FOXA1 is a determinant of multidrug resistance in an E-cadherin independent manner.

### FOXA1 modulates anchorage independence

**Fig. 4:**
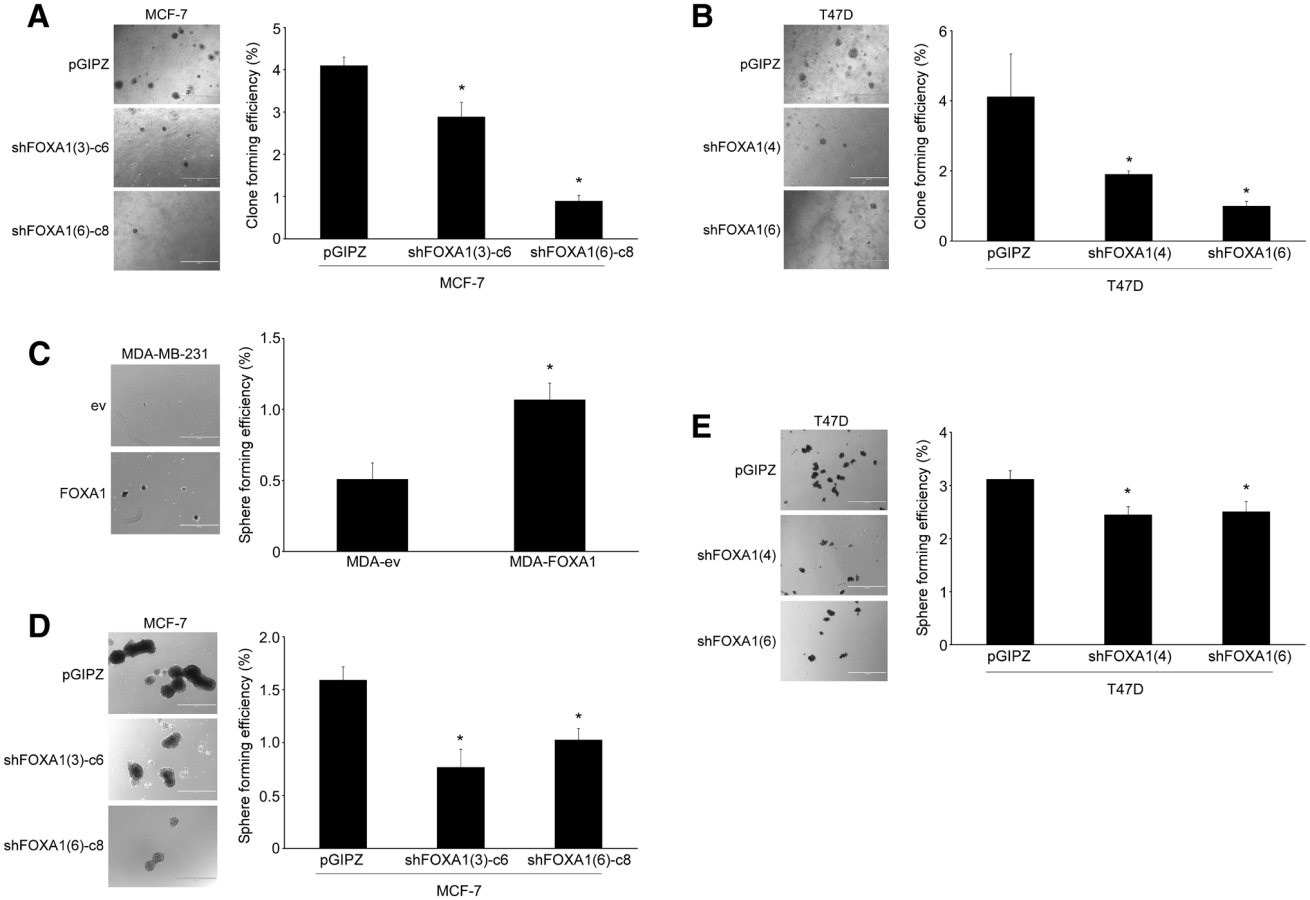
FOXA1 modulates anchorage independence. Anchorage independence growth assays included colony formation in soft agar (**a**, **b**) and mammosphere formation in low-attachment plates (**c**–**e**) and were used as in vitro surrogates for tumor formation in FOXA1-downregulated MCF-7 (**a**, **d**), T47D (**b**, **e**) and FOXA1-overexpressing MDA-MB-231 cells (**c**). **a**, **b** Soft agar assay. Cells (2 × 10^4^/well) were seeded in 6-well plates with medium containing soft agar and the formation of colonies determined after 4 weeks of culture. **c**–**e** Mammosphere assay. Cells (4 × 10^3^/well) were grown in 6-well low-attachment plates for 2 weeks. In all cases, clones or spheres larger than 100 μm in diameter were counted. Clone or sphere forming efficiency calculated relative to the total number of plated cells ×100. Pictorial data show representatives of at least three independent experiments. Numerical data represent the average ± SD of three different experiments (*p < 0.05). Bar represents 1000 μm

Drug resistance is of the outmost importance, especially in a metastatic setting, where secondary tumors have formed, as this dramatically reduces the survival of cancer patients. Anchorage-independent growth is a phenotype acquired by cancer cells especially important for the colonization of secondary sites [8], as it permits the survival of cancer cells in the circulation. Drug resistant cells normally acquire tumor formation capabilities [32] and thus we tested how the experimental modulation of FOXA1 levels affected anchorage independence. For this we used two standard assays, growth in soft agar and in low-attachment plates [33]. Downregulation of FOXA1 in MCF-7 cells led to a decrease in the number of clones in soft agar (Fig. 4a) and mammospheres in liquid cultures (Fig. 4d). Similar data were obtained in FOXA1-downregulated T47D cells (Fig. 4b, e). Conversely, upregulation of FOXA1 in MDA-MB-231 cells led to an increase in mammosphere formation (Fig. 4c). MDA-MB-231 cells did not form robust clones in soft agar assays, a phenomenon already reported [34]. Importantly, *NANOG*, a marker associated with tumor initiation capabilities [35] was upregulated in MDA-FOXA1 cells (Fig. 2c).

In conclusion, FOXA1 is a determinant of multidrug resistance and anchorage independence in breast cancer cells.

## Discussion

During tumor progression, some new characteristics, such as chemotherapy resistance, arise which help its proliferation and survival [21]. Here we describe the role of FOXA1 as a key regulator of multidrug resistance and anchorage independence. Although the regulation of chemotherapy response by FOXA1 has not been addressed before, it is well established that FOXA1 overexpression mediates endocrine resistance in ER-positive breast cancer [18]. FOXA1 modulates a multitude of genes, including the LYPD3/AGR2 receptor-ligand complex, which controls the transition from endocrine therapy-sensitive to -resistant ER-positive breast cancer [36]. In addition, FOXA1 mediates the reprogramming of ER binding in ER-positive disease; it is present in 95% of metastatic samples, and correlates with metastasis [37].

While the role of FOXA1 in resistance to traditional chemotherapy has not been explored in breast cancer, FOXA1 drives cell cycle progression in chondrosarcoma, a malignant bone tumor highly resistant to chemotherapy [38]. Here we present data indicating that FOXA1 regulates doxorubicin and paclitaxel resistance in breast cancer cells. Multidrug resistance, defined as the resistance to structurally and mechanistically dissimilar drugs, is a common phenomenon observed in progressing cancer cells and poses a great obstacle to ensuring good clinical outcomes [28]. Response rates to first-line treatments with paclitaxel or docetaxel in patients with metastatic breast cancer are between 30 and 70% and do not last long, with most patients progressing in as short as 6 months after treatment [39]. An earlier report analyzed transcriptome changes in MCF-7 and T47D cells upon experimental downregulation of FOXA1 by transient transfections with siRNAs [14]. Although this study did not address the cell response to drugs, its gene signatures do not include any of the traditional genes associated with multidrug resistance, such as membrane pumps or apoptotic proteins [40]. Stably transfected cells, like the ones in our study, provide a more robust and reliable phenotype than that obtained after transient transfections, thus warranting further genomic studies with these cell models. Understanding mechanisms behind multidrug resistance can help identify potential treatment targets and, in the long run, improve clinical outcomes for patients with poor prognosis. Pharmacological targeting of ABC transporters modulating multidrug resistance has not reached the clinic despite many years of drug development. Thus, targeting regulators of multidrug resistance may offer another alternative for cancer treatment. Although we do not currently know the genes downstream from FOXA1 which regulate multidrug resistance, our data clearly supports a role of FOXA1 in the regulation of genes controlling response to doxorubicin and paclitaxel in breast cancer cell models.

Another feature associated with cancer progression, acquisition of anchorage independence, is elevated upon experimental FOXA1 overexpression, supporting its role in cancer progression by targeting genes that lead to tumor formation and drug resistance. Using 3D cultures as an in vitro surrogate for tumor formation, we show that FOXA1 promotes anchorage independence, a key property of tumor initiating cells [33]. In FOXA1-overexpressing MDA-MB-231 cells, this was determined by mammosphere formation in liquid cultures. Conversely, downregulation of FOXA1 in MCF-7 and T47D cells led to a decrease in both the number of tumor initiating cells in the mammosphere formation and soft agar assays. Thus, FOXA1 regulates anchorage-independent growth in breast cancer cells.

FOXA1, together with EP300 and RUNX1, is a positive regulator of E-cadherin expression [19]. As loss of E-cadherin is a hallmark of metastasis [41], and experimental downregulation of EP300 leads to a decrease in E-cadherin levels [42], we initially hypothesized that downregulation of FOXA1 levels would lead to a decrease in E-cadherin expression. However, we observed no E-cadherin downregulation upon FOXA1 knockdown in both MCF-7 and T47D cells, suggesting that downregulation of FOXA1 alone is not sufficient to modulate E-cadherin expression. Importantly, FOXA1-depleted cells appear to overcompensate by upregulating the other *CDH1* transcriptional activators, EP300 and RUNX1. As this is not the case upon EP300 downregulation [42], this suggests a certain hierarchy underlying the transcriptional control of E-cadherin.

FOXA1 modulation of anchorage independence and drug sensitivity supports a role in orchestrating programs making breast cancer cells more aggressive. As multidrug resistance is a key determinant of patient survival, the future identification of FOXA1-regulated genes responsible for multidrug resistance will be crucial for the development of targeted therapies and reduction of breast cancer mortality.

## Supplementary Information

Below is the link to the electronic supplementary material.

Supplementary file1 (PDF 118 KB)

## Data Availability

All data generated or analyzed during this study are included in this published article and its supplementary information files.
