# Supplementary material for: FOXA1 is a determinant of drug resistance in breast cancer cells
Source: Breast Cancer Res Treat. 2021 Jan 8;186(2):317–26. doi: 10.1007/s10549-020-06068-5 (PMC7990828; doi:10.1007/s10549-020-06068-5)
Supplement: Supplementary file 1 — Supplementary file1 (PDF 118 KB) [file 10549_2020_6068_MOESM1_ESM.pdf]

Supplementary Information

FOXA1 is a determinant of drug resistance in breast cancer cells

Uttom Kumar, Anastasia Ardasheva[a], Zimam Mahmud[b], R. Charles Coombes and Ernesto Yagüe[c]

Division of Cancer, Imperial College Faculty of Medicine, Hammersmith Hospital Campus, Du Cane Road, London W12 0NN, United Kingdom

[a] Present address Medical Sciences Division, University of Oxford, Oxford, United Kingdom
[b] Present address: Department of Biochemistry and Molecular Biology, University of Dhaka, Dhaka-1000, Bangladesh

[c] Corresponding author: ernesto.yague@imperial.ac.uk

Supplementary Table 1. Oligonucleotides used in this study

Gene	Oligo name	Sequence forward oligo (5'-3')	Oligo name	Sequence reverse oligo (5'-3')
ABCB1	OLEY509	TTCAGGTGGCTCTGGAT	OLEY510	CTGTAGACAAACGATGAGCTATCACA
ABCG2	OLEY321	TGGCTGTCATGGCTTCAGTA	OLEY322	GCCACGTGATTCTTCCACAA
BCL2	OLEY551	GATTGTGGCCTTCTTTGAG	OLEY552	GTTCACAAAGGCATCC
CDH1	OLEY513	GATTCTGCTGCTCTTGCT	OLEY514	GTCAAAGTCCTGGTCCTC
EP300	OLEY529	TATCTTCCATTGCCATCCTC	OLEY530	CCTTGTAGTCATGGACAATAC
ESR1	OLEY678	GGAGTGTACACATTTCTGTC	OLEY679	CAAAGTGTCTGTGATCTTGTC
NANOG	OLEY662	CTATCCATCCTTGCAAATGTC	OLEY663	GTTCTGGTCTTCTGTTTCTTG
RPLP0	OLEY549	GCAGCATCTACAACCCTGAAG	OLEY550	CACTGGCAACATTGCGGAC
RPS14	OLEY373	TCACCGCCCTACACATCAAACT	OLEY374	CTGCGAGTGCTGTCAGAGG
SNAI1	OLEY442	AGGCCATGTCCGGACCCACA	OLEY443	GTGGAGCAGGGACATTCGGGA
SNAI2	OLEY608	CAGTGATTATTTCCCCGTATC	OLEY609	CCCCAAAGATGAGGAGTATC
ZEB1	OLEY409	TTACACCTTTGCATACAGAACCC	OLEY410	TTTACGATTACACCCAGACTGC
ZEB2	OLEY606	ATTTCAGGGAGAATTGCTTG	OLEY607	TGTTCGTATTTATGTCGCAG

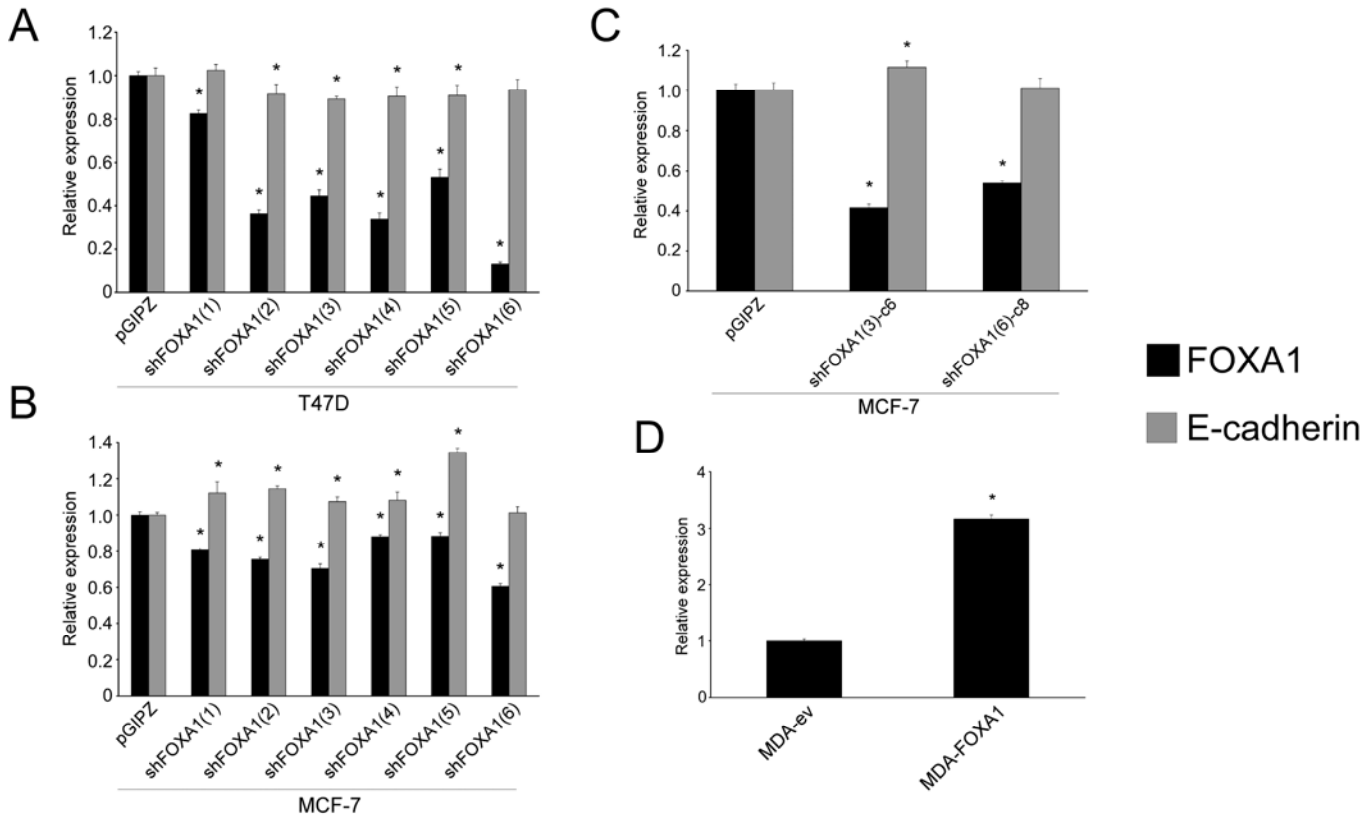

Supplementary Fig. 1 Image quantification of immunoblots shown in Fig. 1-main text. a,b) Stably transfected T47D (a) and MCF-7 (b) cells by expression of *FOXA1* mRNA targeting small hairpins (shFOXA1(1) to shFOXA1(6)). Empty vector pGIPZ was used as a negative control. c) Selected clones from MCF-7 transfected pools. The two clones with the most FOXA1 down-regulation were selected. e) Stably transfected MDA-MB-231 cells by transfection of a FOXA1-expressing plasmid. Empty pcDNA3.1 vector (ev)-transfected cells were used as a control. In all quantifications, FOXA1 and E-cadherin signals were normalized to the β-actin signal. Data represent the average ± SD of three experiments. *, $p < 0.05$ of comparisons to vector controls.
